# Reliability of short-term measurement of heart rate variability on rest in individuals with temporomandibular disorder

**DOI:** 10.1038/s41598-025-00560-y

**Published:** 2025-05-22

**Authors:** Rodrigo Costa Cutrim, Aldair Darlan Santos-de-Araújo, Cassius Iury Anselmo-e-Silva, Janaina de Oliveira Brito Monzani, Rudys Rodolfo de Jesus Tavarez, Maria Cláudia Gonçalves, Etevaldo Matos Maia-Filho, Audrey Borghi-Silva, Almir Vieira Dibai-Filho, Daniela Bassi-Dibai

**Affiliations:** 1 Postgraduate Program in Dentistry, Ceuma University, São Luís, MA Brazil; 2 Department of Physical Therapy, Federal University of São Carlos, São Carlos, SP Brazil; 3 Postgraduate Program in Adult Health, Federal University of Maranhão, São Luís, MA Brazil; 4 Postgraduate Program in Programs Management and Health Services, Ceuma University, Rua Josué Montello, 1, Jardim Renascença, São Luís, MA 65075-120 Brazil

**Keywords:** Autonomic nervous system, Measurement properties, Musculoskeletal diseases, Computational biology and bioinformatics, Data processing, Cardiology, Oral diseases

## Abstract

Individuals with temporomandibular disorder (TMD) experience autonomic dysfunction, which disrupts the balance between the sympathetic and parasympathetic nervous systems. This imbalance can result in altered heart rate variability (HRV) and other physiological disturbances. However, it is important to note that, to date, no research has investigated the inter- and intra-rater reliability of HRV measurements in individuals with TMD. The objective of this investigation was to analyze the intra- and inter-examiner reliability of HRV captured by a polar heart rate monitor in individuals with TMD. The RR interval, the time elapsed between two successive R-waves of the QRS signal on the electrocardiogram (RRi), were recorded during a 10 min period in a supine position using a portable heart rate monitor (Polar® V800 model). The data were transferred into Kubios® HRV standard analysis software and analyzed within the stable sessions containing 5 min sequential RRi. The intraclass correlation coefficient (ICC) ranged from 0.907 to 0.998 according to the intra-examiner analysis by Examiner 1 and 0.875 to 0.998 according to the intra-examiner by Examiner 2. The inter-examiner ICC ranged from 0.796 to 0.996. The coefficient of variation was up to 9.60 for Examiner 1 intra-examiner analysis, 11.95 for Examiner 2 intra-examiner analysis and 14.88 for inter-examiner analysis. The HRV analysis captured by a Polar cardio frequency meter presented adequate reliability when considering different times and examiners. Intra-examiner reliability showed ICC values ranging from 0.842 to 0.999, and inter-examiner reliability ranged from 0.796 to 0.994. Bland–Altman plots indicated good agreement with minimal bias. Coefficients of variation were below 10% for most variables.

## Introduction

Temporomandibular disorder (TMD) can be generically characterized as a dysfunction that, in short, is associated with the temporomandibular joint and the muscles involved in mastication^1^. Noises, deviations during mouth opening, limitations in joint range, difficulty chewing, joint and muscle pain, headache, and tinnitus are among the main signs and symptoms most prevalent among individuals affected by this disorder^2^. Current estimates indicate that approximately 39% of the population presents at least one of the signs or symptoms attributable to TMD and, despite the robustness of the current literature with studies of significant quality, there is still much to be investigated about this condition, especially with regard to the behavior of the autonomic nervous system^3–5^.

The integrity of autonomic nervous function can be assessed noninvasively through heart rate variability (HRV)^6,7^. By definition, HRV is understood as the time fluctuations between the RR intervals of consecutive heartbeats, directly reflecting the behavior and function of the sinus node^8–10^. Its usefulness and predictive capacity as a health biomarker have been previously explained^9^ and, in summary and in a didactic manner, an adequate adaptation of the cardiovascular system is reflected in a higher HRV, indicating a better adaptive capacity of this system to intrinsic and extrinsic change processes such as stress, physical exercise, use of medications, physical conditioning, frights, fear and escape situations^8–10^. In turn, a deteriorated adaptation in the cardiovascular system is reflected in a lower HRV and, consequently, in a greater autonomic dysfunction. In individuals with TMD, there is a limitation of studies that endorse this outcome, however, previous studies have proven that this population suffers from reduced HRV and that nocturnal HRV is strictly lower when compared to healthy individuals^5,11,12^.

The need to broadly understand autonomic dysfunction in these individuals has led researchers and clinicians to include HRV and use metrics from this outcome in their assessments^13,14^. However, a curious fact is that to date, no investigation has been conducted to verify the inter- and intra-rater reliability of HRV assessment in individuals with TMD^11–13^. In this regard, important guidelines on measurement properties not only consider this type of investigation to be part of the instrument reliability process, but also highlight its impact on the quality of the results presented when assessing different populations^13,14^.. We know that biological signal processing and HRV analysis can be easily biased by factors that may be associated with the individual nature of each evaluator^15,16^. Currently, there is no consensus on how this measurement should be analyzed, and the evaluator-dependent decision of the best time window for interpreting the results and, consequently, understanding the ANS, can compromise the analyses and generate inaccurate results that do not reflect reality^15,16^. Within this context and taking as a hypothesis the idea that short-term HRV assessed by a heart rate monitor may present adequate reliability values when considering different examiners and a wash-out period, we aimed to investigate intra- and inter-examiner reliability of HRV in individuals with TMD.

## Methodology

### Study design

This reliability study was conducted in accordance with the Guidelines for Reporting Reliability and Agreement Studies (GRRAS)^14^. The research was conducted at Ceuma University, in São Luís, MA, Brazil, after approval by the Research Ethics Committee of Ceuma University (protocol number: 5.674.373) and followed the principles established in the Declaration of Helsinki. Participants were fully informed about the objectives of the study and provided their written informed consent.

### Participants

Adult individuals aged between 18 and 55 years with myogenic TMD confirmed by the Fonseca Anamnestic Index (IAF) were included. The exclusion criteria were: clinical diagnosis of rheumatic, cardiovascular, metabolic, respiratory, or systemic neuromuscular diseases, use of total or partial prostheses, history of trauma to the face and/or temporomandibular joint, joint dislocation, medication treatment that affects the musculoskeletal system (such as analgesics, anti-inflammatories, and muscle relaxants), as well as any sign of malignant tumor, inflammatory or infectious disease, diagnosis of fibromyalgia, or any other condition that would prevent the evaluations proposed in this study.

#### R-R intervals recordings: heart rate variability

Before collecting biological signals for heart rate variability (HRV) analysis, patients were given instructions 24 h before the examination. The guidelines included abstaining from alcohol, caffeine, nicotine, chocolate, soft drinks and energy drinks, avoiding intense physical exercise and ensuring a good night’s sleep the day before and on the day of the examination. To control for the influence of the circadian rhythm, the assessments were performed in the morning, in a quiet environment, with room humidity and temperature maintained between 50–60% and 20–24 °C, respectively^17^. Patients remained in the supine position for approximately 10 min to stabilize the heart rate after changing position. Then, R-R intervals were recorded with a Polar S810i heart rate monitor (Polar Electro, Kempele, Oulu, Finland) and the data were transferred to a computer using Polar Advantage software (Kempele, Oulu, Finland) for HRV analysis.

### Heart rate variability analysis and data processing

Heart rate variability (HRV) data analysis was performed using Kubios HRV software (MATLAB, version 3.5, Kuopio, Finland)^18^. This software provides a user-friendly interface for HRV analysis, with options for time, frequency, and nonlinear domain analysis^9,19^. Data were examined in stable 5-min sessions (short-term HRV analysis), and the segment with the greatest signal stability was selected^19^. Signal quality was checked visually; if more than 10% of the beats were ectopic in relation to the total pure sinus beats, the data were discarded. To identify the longest period of stability, the following criteria were followed: (1) absence of large outliers in the R-R intervals (i.e., intervals much larger or smaller than the mean, as visually inspected by the researcher on the HRV recording); (2) equidistance of R-R intervals; and (3) Gaussian distribution of R-R intervals and heart rate curves^19,20^.

HRV was analyzed by linear statistical measures (time and frequency domain) and by non-linear statistical measures. With regard to the linear analysis in the time domain, the standard deviation of all normal N–N intervals (SDNN) in ms, the square root of successive mean squared differences of RR (RMSSD) in ms and the number of interval differences of successive NN intervals greater than 50 ms divided by the total number of NN intervals (pNN50) in percentage, the first representing global HRV and the last two representing parasympathetic modulation^9,19^. For the linear analysis in the frequency domain, the spectral analysis was performed by the Fast Fourier Transform and the components were reported in high frequency (HF) in absolute values (ms^2^) and in normalized units (n.u) (0.15–0. 4 Hz), which reflects vagal modulation, and low frequency (LF) in absolute values (ms^2^) and in normalized units (n.u) (0.04–0.15 Hz), which have been predominantly related to high sympathetic modulation and low parasympathetic modulation^9,19^.

Nonlinear analysis of HRV was performed to obtain the standard deviation perpendicular to the line of identity (SD1), plot the standard deviation along the line of identity (SD2) (representing parasympathetic and sympathetic modulation, respectively) and HRV fluctuation analysis. Trend that describes short (DFα1) and long-term fluctuations (DFα2) where the value one (1) indicates chaotic behavior, one and a half (1.5) corresponds to regularity and a half (0.5) corresponds to randomness^9,21,22^.

### Statistical analysis

Intraclass correlation coefficient (ICC)_2,1_^23^, confidence interval (95% IC), standard error of measurement (SEM) and minimum detectable change (MDC), coefficient of variation (CV) and Bland–Altman plots (mean difference [bias] and 95% limits of agreement[upper and lower]) were used for the analysis of the intra- and inter-examiner reliability of the HRV variables. The results from the ICC_2,1_ analysis were interpreted according to the Fleiss study: values below 0.40, reliability was considered low; between 0.40 and 0.75, moderate; between 0.75 and 0.90, substantial; and finally, for values greater than 0.90, reliability was rated as excellent. SEM was calculated using the formula: $$standard deviation of means X \surd (1-ICC)$$^24,25^. MDC was calculated using the formula: $$1.96\times SEM\times \sqrt{2}$$^24,25^. The CV is the ratio of the standard deviation to the mean value and represents the extent of variability of an assay. It is expressed as a percentage of deviation from the mean; the larger the CV, the greater the error in the assay. The formula for computation of CV is straight forward: CV(%) = $$\left(\frac{standard deviation}{mean}\right)X 100$$^26^. The Bland Altman Plots were used as an alternative analysis, based on the quantification of the agreement between two quantitative measurements by studying the mean difference and constructing limits of agreement. The midline represents the mean systematic difference between inter and intra-examiner scores ($$\overline{d }$$) and can be interpreted as being bias estimated by the mean difference between the two measures (X1-X2)/n, where "n" represents the number of individuals included in the sample. The two dotted lines above and below the line mean represent the limits of agreement, and these are drawn at $$\overline{d }$$ ± 1.96 × s, where “s” represents standard deviation of the differences^27,28^. All analyzes were performed using GraphPad Prism software (version 8.0.1 for Windows, GraphPad Software, San Diego, California USA). The probability of type 1 error occurrence was established at 5% for all tests (*p* < 0.05).

## Results

**Fig. 1:**
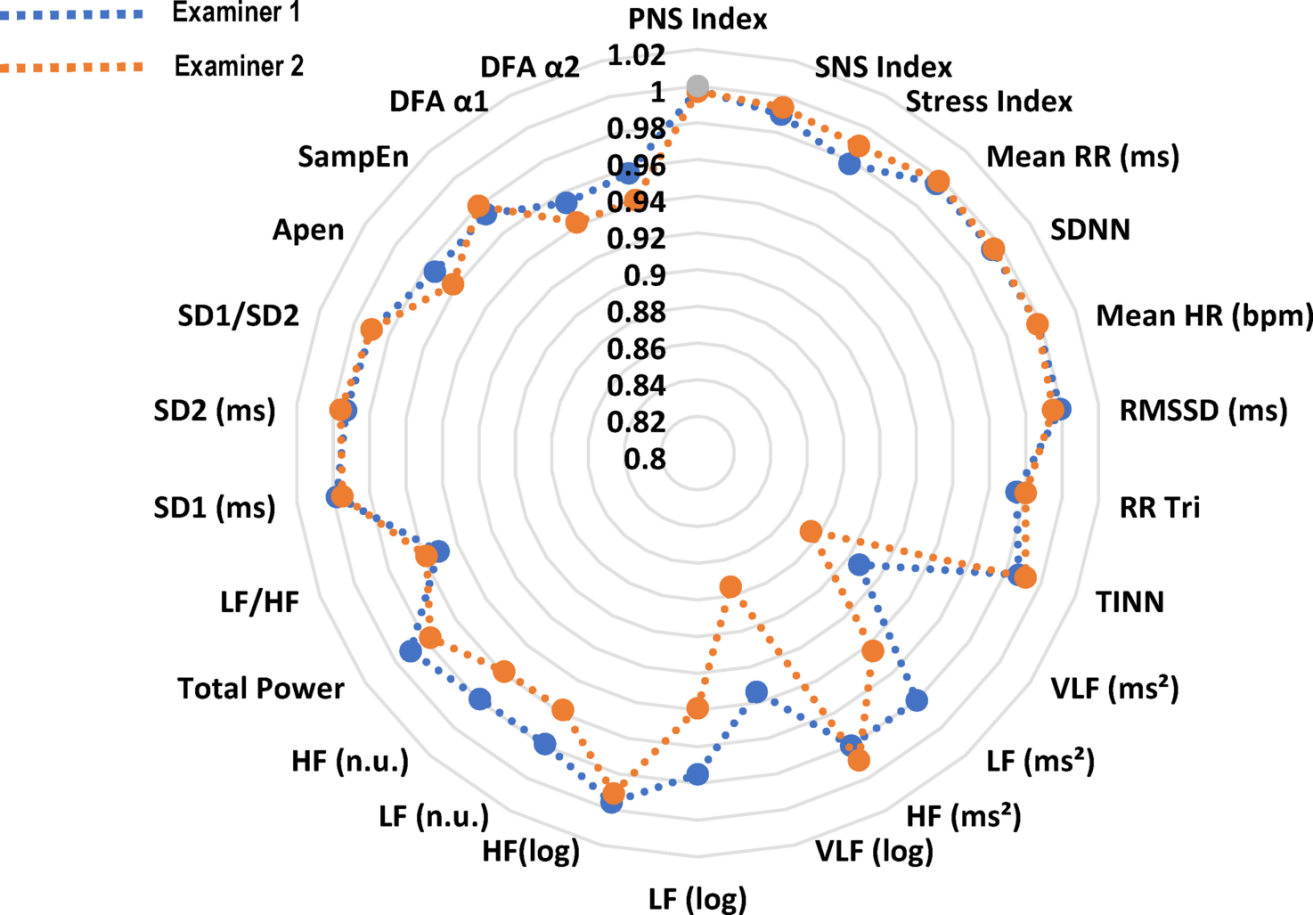
Radar chart illustrating intra-examiner intraclass correlation coefficients. RR: interbeat intervals between all successive heartbeats; PNS: parasympathetic nervous system; SNS: sympathetic nervous system; ms: milliseconds; SDNN: standard deviation of the N–N interval; bpm: beats per minute; RMSSD: root mean square differences of successive RR intervals; RR Tri: integral of the RR intervals histogram divided by the height of the histogram; TINN: baseline width of the RR intervals histogram; VLF: very-low frequency; LF: low-frequency band; HF: the high-frequency band; ms^2^: milliseconds squared; n.u: normalized units; log: logarithmic; LF/HF: ratio of LF-to-HF; SD1: Poincaré plot standard deviation perpendicular the line of identity; SD2: Poincaré plot standard-deviation along the line of identity; SD2/SD1: ratio of SD1-to-SD2; ApEn: approximate entropy; SampEn: sample entropy; DFA α1: detrended fluctuations analysis, which describes short-term fluctuations; DFA α2: detrended fluctuation analysis, which describes long-term fluctuations.

**Fig. 2:**
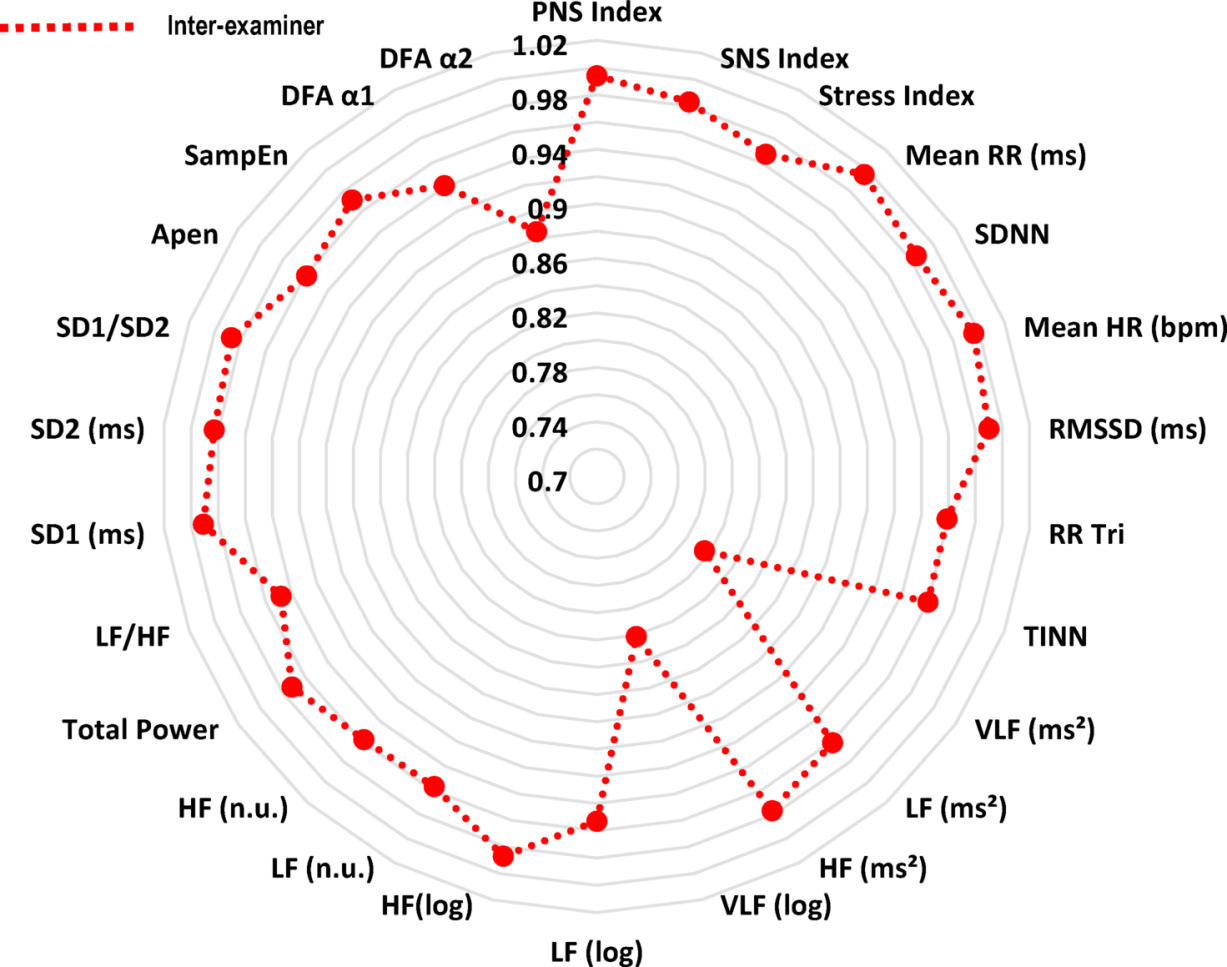
Radar chart illustrating inter-examiner intraclass correlation coefficients. RR: interbeat intervals between all successive heartbeats; PNS: parasympathetic nervous system; SNS: sympathetic nervous system; ms: milliseconds; SDNN: standard deviation of the N–N interval; bpm: beats per minute; RMSSD: root mean square differences of successive RR intervals; RR Tri: integral of the RR intervals histogram divided by the height of the histogram; TINN: baseline width of the RR intervals histogram; VLF: very-low frequency; LF: low-frequency band; HF: the high-frequency band; ms^2^: milliseconds squared; n.u: normalized units; log: logarithmic; LF/HF: ratio of LF-to-HF; SD1: Poincaré plot standard deviation perpendicular the line of identity; SD2: Poincaré plot standard-deviation along the line of identity; SD2/SD1: ratio of SD1-to-SD2; ApEn: approximate entropy; SampEn: sample entropy; DFA α1: detrended fluctuations analysis, which describes short-term fluctuations; DFA α2: detrended fluctuation analysis, which describes long-term fluctuations.

The sample size calculation was performed based on the Fleiss^29^ study. Assuming a minimum acceptable ICC of 0.40 and an expected ICC of 0.75 for moderate reliability, together with an alpha error of 5%, a statistical power of 80% and a sample loss rate of 15%, it was estimated that at least 33 participants would be needed. A total of 44 individuals were initially selected for the study. After checking the inclusion criteria, 8 participants were excluded. Thus, the final sample comprised 36 people, including 10 men (27%) and 26 women (73%). Information on the clinical and anthropometric characteristics of the participants is listed in Table 1. Table 2 shows the mean values and standard deviations of heart rate variability observed at different evaluation times by each examiner (Figs. 1 and 2).

**Table 1:** Personal and clinical characteristics of the study sample.

Variables	Mean (SD)
Age (years)	34.25 ± 8.64
Gender	
Male n (%)	10 (27)
Female n (%)	26 (73)
Weight (kg)	70.50 ± 13.85
Height (m)	1.63 ± 0.08
BMI (kg/m^2^)	25.98 ± 3.60
Pain-AVS	6 ± 1
Fonseca Anamnestic Index	55.00 ± 11.28
Respiratory rate at rest (min)	16 ± 3

*SD* standard deviation, *BMI* body mass index, *kg* kilos, *m* meter, *AVS* analogic visual scale.

**Table 2:** Mean values and standard deviation (SD) of HRV in individuals with TMD in the supine position.

HRV indices	Examiner 1	Examiner 1	Examiner 2	Examiner 2
	Test	Retest	Test	Restest
Overview				
PNS Index	− 1.14 ± 0.73	− 1.16 ± 0.68	− 1.14 ± 0.71	− 1.16 ± 0.69
SNS Index	1.78 ± 1.06	1.73 ± 1.03	1.72 ± 1.00	1.74 ± 0.99
Stress Index	15.05 ± 4.90	14.64 ± 4.63	14.66 ± 4.50	14.71 ± 4.38
Time domain				
Mean RR (ms)	764.07 ± 87.60	761.60 ± 82.52	764.30 ± 85.95	763.11 ± 84.47
SDNN (ms)	31.77 ± 12.74	32.39 ± 12.71	32.05 ± 12.27	31.88 ± 12.14
Mean HR (bpm)	79.48 ± 8.68	79.66 ± 8.47	79.44 ± 8.66	79.53 ± 8.58
RMSSD (ms)	26.84 ± 14.14	26.91 ± 14.02	26.91 ± 13.82	26.48 ± 13.57
pNN50%	8.21 ± 11.91	6.42 ± 7.68	6.51 ± 8.02	7.87 ± 11.56
RR Tri	8.13 ± 3.09	8.40 ± 3.45	8.39 ± 3.24	8.24 ± 3.03
TINN	156.76 ± 72.60	162.30 ± 64.74	164.43 ± 69.19	159.91 ± 63.26
Frequency domain				
VLF (ms^2^)	83.95 ± 72.60	81.15 ± 63.43	81.95 ± 67.16	82.82 ± 75.33
LF (ms^2^)	636.82 ± 538.66	629.44 ± 488.21	629.18 ± 477.31	582.72 ± 468.06
HF (ms^2^)	390.91 ± 496.16	386.44 ± 563.67	388.48 ± 475.62	387.62 ± 479.04
VLF (log)	4.04 ± 0.93	4.07 ± 0.83	4.11 ± 0.77	4.06 ± 0.86
LF (log)	6.06 ± 0.94	6.11 ± 0.86	6.15 ± 0.78	6.07 ± 0.78
HF (log)	5.35 ± 1.17	5.32 ± 1.15	5.37 ± 1.15	5.35 ± 1.16
LF (n.u.)	64.98 ± 18.49	66.25 ± 18.83	66.40 ± 17.78	64.67 ± 18.47
HF (n.u.)	34.97 ± 18.49	33.69 ± 18.82	33.57 ± 17.77	35.27 ± 18.44
Total power	1112.04 ± 940.20	1097.60 ± 940.85	1099.90 ± 882.43	1053.53 ± 895.37
LF/HF	2.96 ± 2.72	3.20 ± 2.94	3.10 ± 2.77	2.99 ± 2.89
Nolinear methods				
SD1 (ms)	19.01 ± 10.01	18.99 ± 9.70	19.12 ± 10.04	18.75 ± 9.61
SD2 (ms)	40.40 ± 15.80	41.41 ± 15.88	40.82 ± 14.93	40.72 ± 15.01
SD2/SD1	2.31 ± 0.74	2.36 ± 0.71	2.34 ± 0.70	2.37 ± 0.75
Apen	1.17 ± 0.09	1.18 ± 0.09	1.18 ± 0.09	1.18 ± 0.09
SampEn	1.67 ± 0.28	1.66 ± 0.27	1.67 ± 0.26	1.66 ± 0.25
DFA α1	1.17 ± 0.26	1.20 ± 0.27	1.18 ± 0.25	1.18 ± 0.26
DFA α2	0.47 ± 0.15	0.48 ± 0.14	0.48 ± 0.14	0.49 ± 0.16

*HRV* heart rate variability, *SD* standard desviation, *RR* interbeat intervals between all successive heartbeats, *PNS* parasympathetic nervous system, *SNS* sympathetic nervous system, *ms* milliseconds, *SDNN* standard deviation of the N–N interval, *bpm* beats per minute, *RMSSD* root mean square differences of successive RR intervals, *RR Tri* integral of the RR intervals histogram divided by the height of the histogram, *TINN* baseline width of the RR intervals histogram, *VLF* very-low frequency, *LF* low-frequency band, *HF* the high-frequency band, *ms*^2^: milliseconds squared, *n.u* normalized units, *log* logarithmic, *LF/HF* ratio of LF-to-HF, *SD1* Poincaré plot standard deviation perpendicular the line of identity, *SD2* Poincaré plot standard-deviation along the line of identity, *SD2/SD1* ratio of SD1-to-SD2, *ApEn* approximate entropy, *SampEn* sample entropy, *DFA α1* detrended fluctuations analysis. Which describes short-term fluctuations, *DFA α2* detrended fluctuation analysis. Which describes long-term fluctuations.

**Fig. 3:**
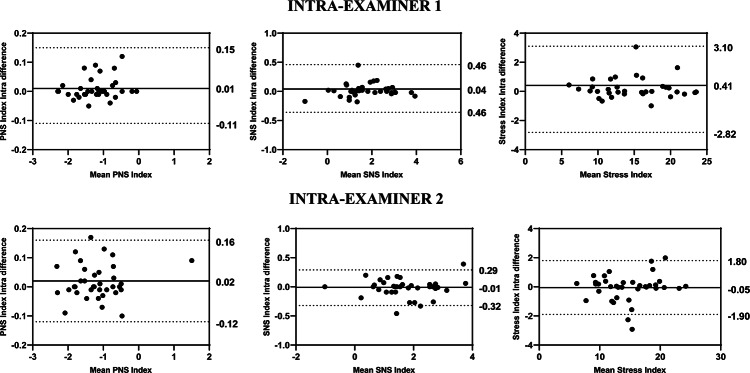
Bland–Altman plots of the intra-examiner variables from the HRV overview. Bland–Altman plots (mean difference [bias] and 95% limits of agreement [upper and lower]). HRV: heart rate variability; PNS: parasympathetic nervous system; SNS: sympathetic nervous system.

**Fig. 4:**
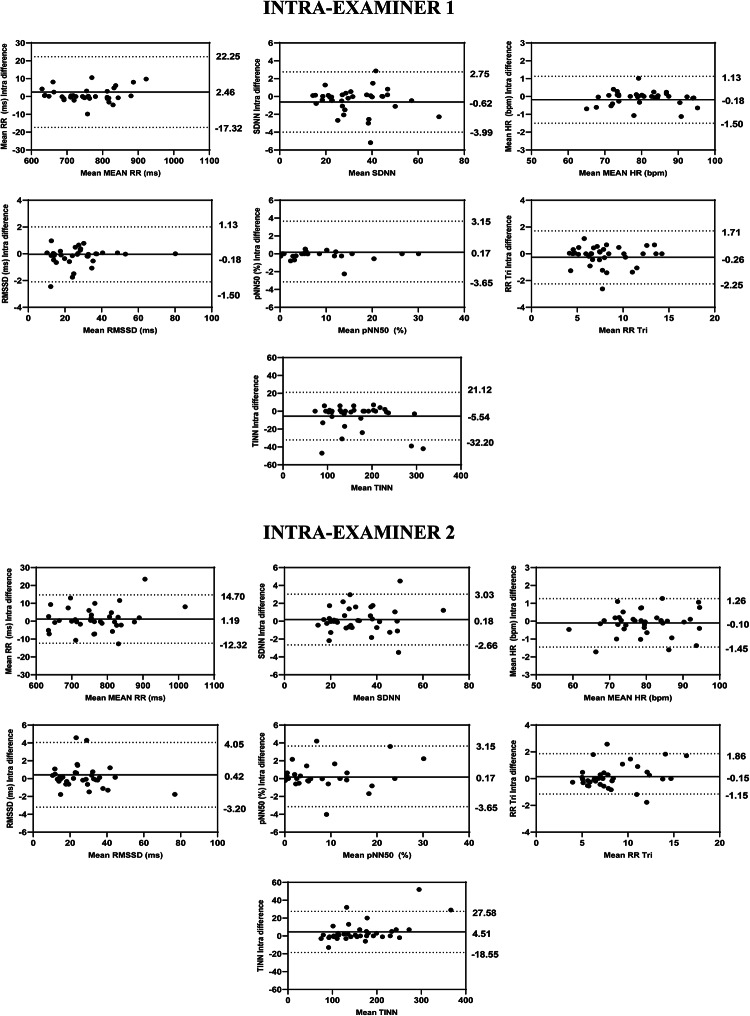
Bland–Altman plots of the intra-examiner variables from the time domain of HRV. Bland–Altman plots (mean difference [bias] and 95% limits of agreement [upper and lower]). HRV: heart rate variability; VLF: very-low frequency; LF: low-frequency band; HF: the high-frequency band; ms^2^: milliseconds squared; n.u: normalized units; log: logarithmic; LF/HF: ratio of LF-to-HF.

**Fig. 5:**
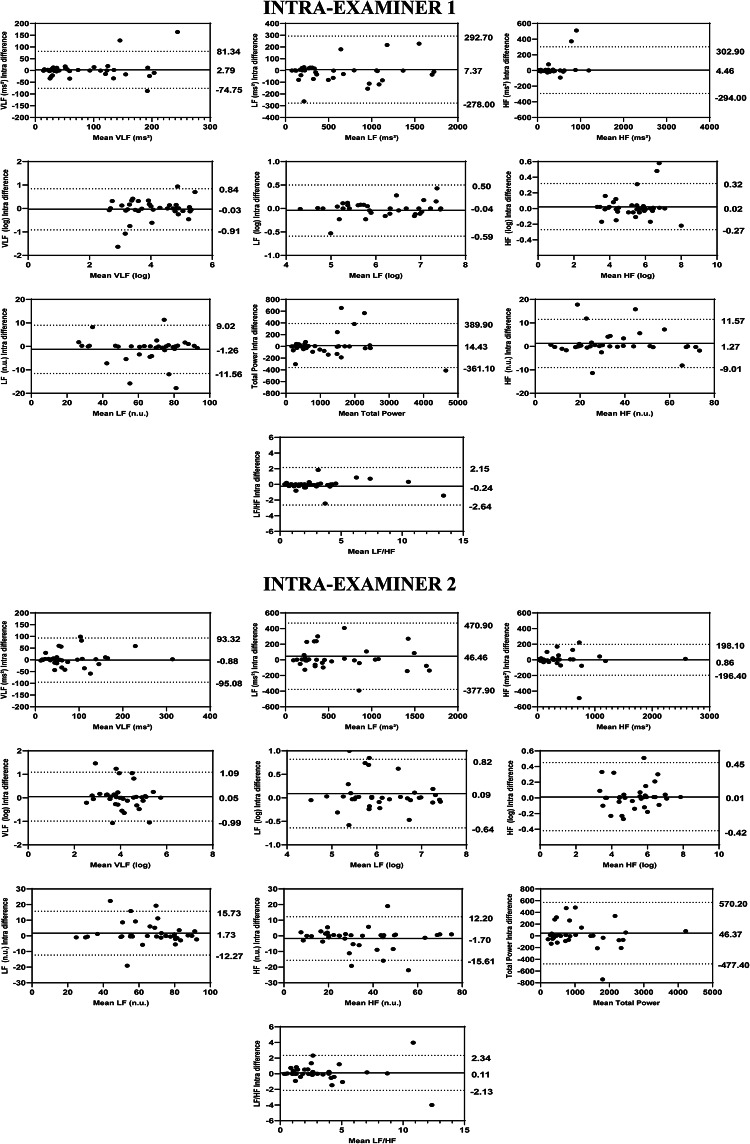
Bland–Altman plots of the intra-examiner variables from the time domain of HRV. Bland–Altman plots (mean difference [bias] and 95% limits of agreement [upper and lower]). HRV: heart rate variability; VLF: very-low frequency; LF: low-frequency band; HF: the high-frequency band; ms^2^: milliseconds squared; n.u: normalized units; log: logarithmic; LF/HF: ratio of LF-to-HF.

**Fig. 6:**
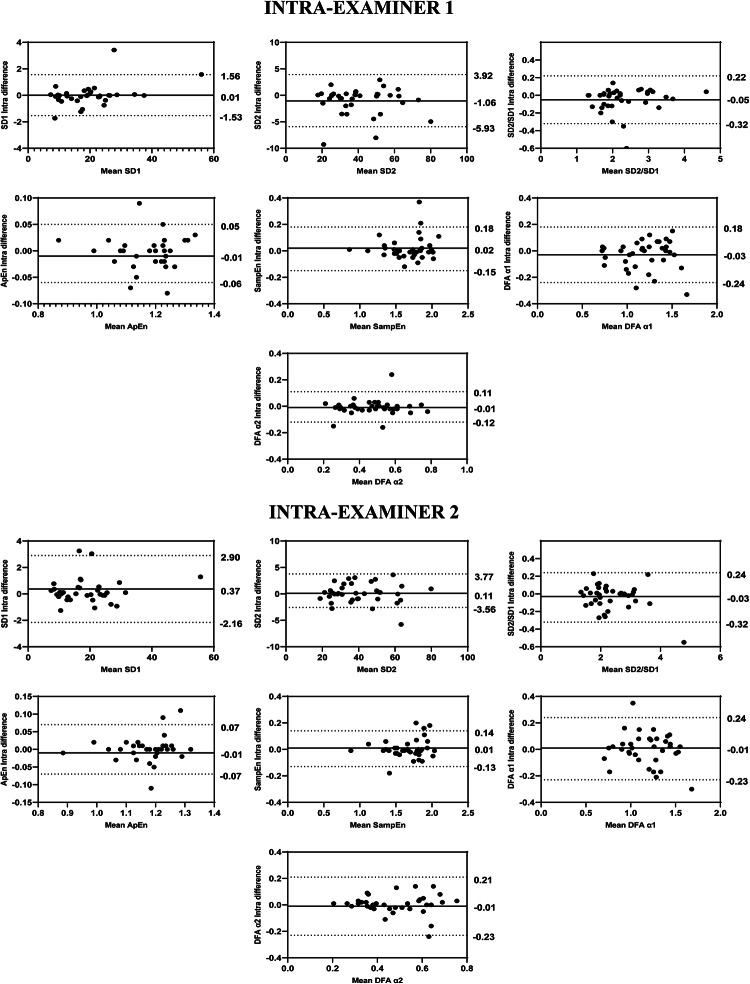
Bland–Altman plots of the intra-examiner variables from the nonlinear analysis of HRV**.** Bland–Altman plots (mean difference [bias] and 95% limits of agreement [upper and lower]). HRV: heart rate variability; SD1: Poincaré plot standard deviation perpendicular the line of identity; SD2: Poincaré plot standard-deviation along the line of identity; SD2/SD1: ratio of SD1-to-SD2; ApEn: approximate entropy; SampEn: sample entropy; DFA α1: detrended fluctuations analysis, which describes short-term fluctuations; DFA α2: detrended fluctuation analysis, which describes long-term fluctuations.

The intra-examiner reliability results for Examiner 1 indicated that the ICCs for the HRV variables ranged from 0.978 to 0.998 for the overall analysis, from 0.987 to 0.999 for the time domain, from 0.907 to 0.996 for the frequency domain, and from 0.954 to 0.998 for the nonlinear methods (Fig. 1). The SEM ranged from − 2.75 to 4.76% in the overall analysis, from 0.48% to 6.26% in the time domain, from 1.38 to 25.13% in the frequency domain, and from 1.24 to 6.33% in the nonlinear methods. The MDC ranged from − 7.60 to 16.50% in the general analysis, from 1.95 to 17.34% in the time domain, from 3.81 to 66.95% in the frequency domain, and from 3.42 to 17.55% in the nonlinear methods (Table 3). The Bland–Altman plots showed good intra-examiner agreement for the HRV variables. In the overview (Fig. 3), the highest bias was observed in the Stress Index variable (0.41). In the time domain (Fig. 4), the biases were small, with greater variation in Mean RR (2.46 ms) and TINN (− 5.54 ms). In the frequency domain (Fig. 5), Total Power (14.43 ms^2^) showed the greatest bias dispersion, while in the nonlinear analysis (Fig. 6), the biases were minimal, with SD2 (− 1.06 ms) exhibiting the highest variability.

**Table 3:** Intra-rater reliability of HRV analysis of patients with TMD in the supine position (Examiner 1).

HRV Index	SEM	SEM (%)	MDC	MDC (%)	CV (%)
Overview					
PNS Index	0.03	− 2.74	0.09	− 7.60	− 0.80
SNS Index	0.10	5.95	0.29	16.50	2.74
Stress Index	0.71	4.76	1.96	13.20	1.54
Time domain					
Mean RR (ms)	5.38	0.71	14.91	1.95	0.18
SDNN	0.90	2.80	2.49	7.77	1.46
Mean HR (bpm)	0.38	0.48	1.06	1.34	0.18
RMSSD (ms)	0.45	1.58	1.23	4.37	0.89
RR Tri	0.52	6.26	1.43	17.34	2.69
TINN	7.83	4.91	21.70	13.60	1.91
Frequency domain					
VLF (ms^2^)	20.74	25.13	57.49	66.95	9.60
LF (ms^2^)	72.61	11.47	201.27	31.79	4.95
HF (ms^2^)	79.94	19.28	207.73	53.34	3.10
VLF (log)	0.23	5.58	0.63	15.45	2.76
LF (log)	0.14	2.34	0.39	6.48	0.90
HF(log)	0.07	1.38	0.20	3.81	0.58
LF (n.u.)	2.70	4.12	7.49	11.42	1.80
HF (n.u.)	2.70	7.87	7.49	21.83	3.77
Total power	94.05	8.51	260.70	23.60	4.18
LF/HF	0.63	20.34	1.74	56.38	5.46
Nolinear methods					
SD1 (ms)	0.44	2.33	1.22	6.45	0.92
SD2 (ms)	1.33	3.24	3.67	8.98	1.67
SD1/SD2	0.07	3.10	0.20	8.61	1.50
Apen	0.01	1.24	0.04	3.42	0.63
SampEn	0.04	2.66	0.12	7.38	1.16
DFA α1	0.06	4.80	0.16	13.29	2.54
DFA α2	0.03	6.33	0.08	17.55	2.92

*HRV* heart rate variability, *SEM* standard error measurement, *MDC* minimal detectable change, *SD* standard desviation, *CV* coefficient of variation, *RR* interbeat intervals between all successive heartbeats, *PNS* parasympathetic nervous system, *SNS* sympathetic nervous system, *ms* milliseconds, *SDNN* standard deviation of the N–N interval, *bpm* beats per minute, *RMSSD* root mean square differences of successive RR intervals, *RR Tri* integral of the RR intervals histogram divided by the height of the histogram, *TINN* baseline width of the RR intervals histogram, *VLF* very-low frequency, *LF* low-frequency band, *HF* the high-frequency band, *ms*^2^: milliseconds squared, *n.u* normalized units, *log* logarithmic, *LF/HF* ratio of LF-to-HF, *SD1* Poincaré plot standard deviation perpendicular the line of identity, *SD2* Poincaré plot standard-deviation along the line of identity, *SD2/SD1* ratio of SD1-to-SD2, *ApEn* approximate entropy, *SampEn* sample entropy, *DFA α1* detrended fluctuations analysis. Which describes short-term fluctuations, *DFA α2* detrended fluctuation analysis. Which describes long-term fluctuations.

Regarding the intra-examiner reliability results of the Examiner 2 demonstrated that the ICCs of HRV variables in overview ranged from 0.989 to 0.997, in time-domain ranged from 0.980 to 0.998, in frequency-domain ranged 0.875 to 0.991, while in the nonlinear methods ranged 0.942 to 0.946 (Fig. 1). The SEM ranged from − 3.33 to 4.46% in overview, 0.50 to 5.33% in the time-domain, 2.04 to 30.57% in the frequency-domain and 1.49 to 12.29% in the nonlinear methods. The MDC ranged from − 9.24 to 12.35% in overview, 1.34 to 14.78% in the time-domain, 5.67 to 51.17% in the frequency-domain and 4.12 to 34.08% in the nonlinear methods (Table 4). The Bland–Altman plots demonstrated consistent intra-examiner reliability for the HRV variables. The Stress Index (Fig. 3) from the overview showed a bias of − 0.05 ms. In the time domain (Fig. 4), the biases were small, with greater variation observed in Mean RR (1.19 ms) and TINN (4.51 ms). In the frequency domain (Fig. 5), the variables LF (46.46 ms^2^) and Total Power (46.37 ms^2^) exhibited more considerable dispersion in the limits of agreement. In the nonlinear analysis (Fig. 6), biases were minimal, with SD1 (0.37 ms) showing the most variability.

**Table 4:** Intra-rater reliability of HRV analysis of patients with TMD in the supine position (Examiner 2).

HRV Index	SEM	SEM (%)	MDC	MDC (%)	CV (%)
Overview					
PNS Index	0.04	− 3.33	0.11	− 9.24	− 4.81
SNS Index	0.08	4.46	0.21	12.35	3.52
Stress Index	0.47	3.17	1.29	8.79	1.69
Time domain					
Mean RR (ms)	3.81	0.50	10.56	1.38	0.23
SDNN	0.77	2.41	2.14	6.69	1.22
Mean HR (bpm)	0.39	0.48	1.07	1.34	0.23
RMSSD (ms)	0.97	3.63	2.68	10.06	1.39
RR Tri	0.44	5.33	1.23	14.78	2.64
TINN	6.28	3.87	17.41	10.74	1.45
Frequency domain					
VLF (ms^2^)	25.19	30.57	69.82	84.75	11.95
LF (ms^2^)	111.86	18.46	310.05	51.17	8.27
HF (ms^2^)	50.06	12.90	138.77	35.76	5.03
VLF (log)	0.28	6.80	0.77	18.83	3.27
LF (log)	0.19	3.15	0.53	8.74	1.45
HF(log)	0.11	2.04	0.30	5.67	1.00
LF (n.u.)	3.71	5.67	10.30	15.71	2.73
HF (n.u.)	3.67	10.65	10.16	29.52	4.98
Total Power	134.81	12.52	373.67	34.70	6.41
LF/HF	0.58	19.05	1.61	52.80	7.56
Nolinear methods					
SD1 (ms)	0.69	3.67	1.93	10.17	1.39
SD2 (ms)	0.95	2.32	2.62	6.44	1.31
SD1/SD2	0.07	3.08	0.20	8.53	1.57
Apen	0.02	1.49	0.05	4.12	0.65
SampEn	0.05	2.99	0.14	8.28	1.05
DFA α1	0.06	5.20	0.17	14.43	2.84
DFA α2	0.06	12.29	0.17	34.08	4.34

*HRV* heart rate variability, *SEM* standard error measurement, *MDC* minimal detectable change, *SD* standard desviation, *CV* coefficient of variation, *RR* interbeat intervals between all successive heartbeats, *PNS* parasympathetic nervous system, *SNS* sympathetic nervous system, *ms* milliseconds, *SDNN* standard deviation of the N–N interval, *bpm* beats per minute, *RMSSD* root mean square differences of successive RR intervals, *RR Tri* integral of the RR intervals histogram divided by the height of the histogram, *TINN* baseline width of the RR intervals histogram, *VLF* very-low frequency, *LF* low-frequency band, *HF* the high-frequency band, *ms*^2^: milliseconds squared, *n.u* normalized units, *log* logarithmic, *LF/HF* ratio of LF-to-HF, *SD1* Poincaré plot standard deviation perpendicular the line of identity, *SD2* Poincaré plot standard-deviation along the line of identity, *SD2/SD1* ratio of SD1-to-SD2, *ApEn* approximate entropy, *SampEn* sample entropy, *DFA α1* detrended fluctuations analysis. Which describes short-term fluctuations, *DFA α2* detrended fluctuation analysis. Which describes long-term fluctuations.

**Fig. 7:**
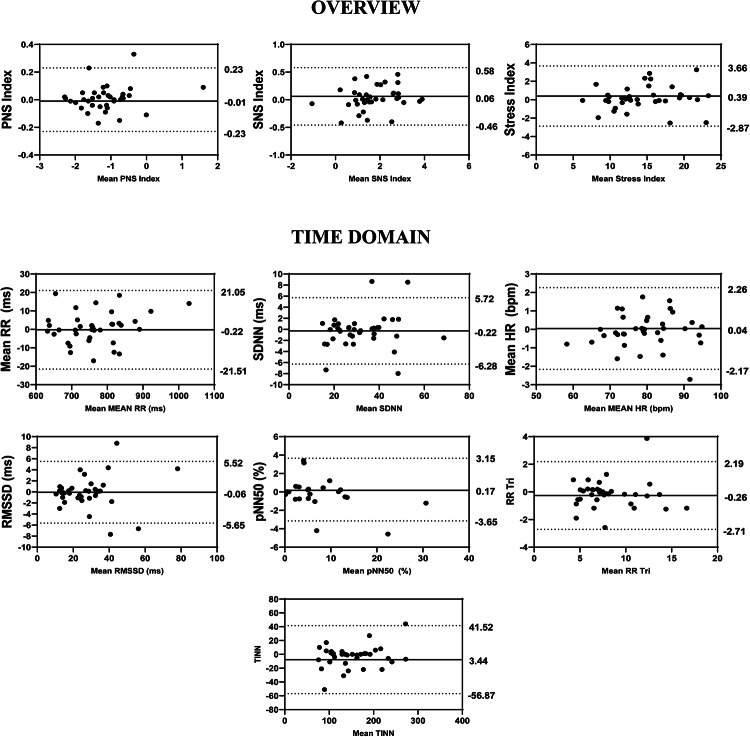
Bland–Altman plots of the inter-examiner variables from the overview and time domain of HRV. Bland–Altman plots (mean difference [bias] and 95% limits of agreement [upper and lower]). HRV: heart rate variability; PNS: parasympathetic nervous system; SNS: sympathetic nervous system; SDNN: standard deviation of the N–N interval; bpm: beats per minute; RMSSD: root mean square differences of successive RR intervals; RR Tri: integral of the RR intervals histogram divided by the height of the histogram; TINN: baseline width of the RR intervals histogram.

**Fig. 8:**
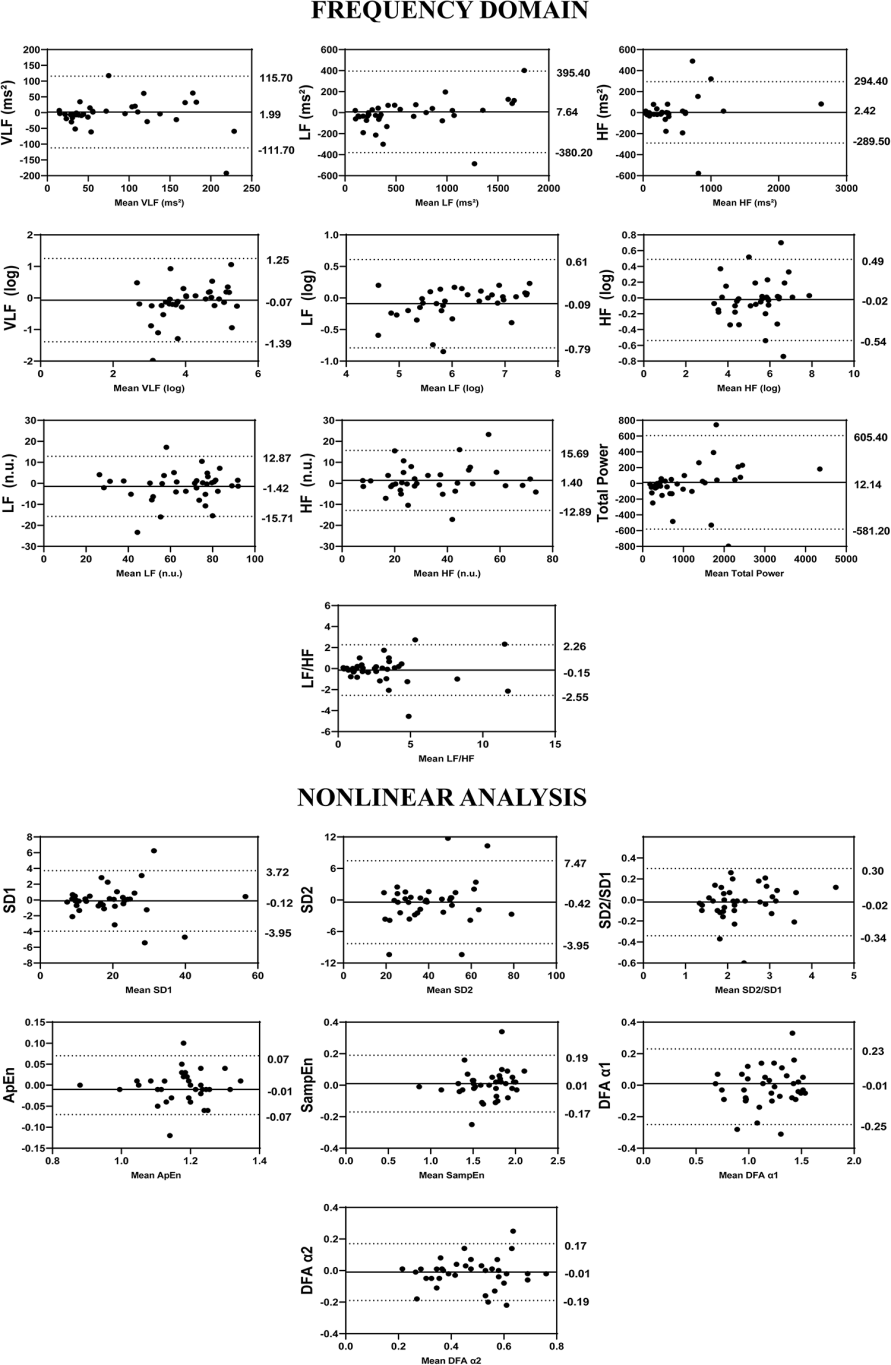
Bland–Altman plots of the inter-examiner variables from the frequency domain and nonlinear analysis. Bland–Altman plots (mean difference [bias] and 95% limits of agreement [upper and lower]). HRV: heart rate variability; VLF: very-low frequency; LF: low-frequency band; HF: the high-frequency band; ms^2^: milliseconds squared; n.u: normalized units; log: logarithmic; LF/HF: ratio of LF-to-HF; SD1: Poincaré plot standard deviation perpendicular the line of identity; SD2: Poincaré plot standard-deviation along the line of identity; SD2/SD1: ratio of SD1-to-SD2; ApEn: approximate entropy; SampEn: sample entropy; DFA α1: detrended fluctuations analysis, which describes short-term fluctuations; DFA α2: detrended fluctuation analysis, which describes long-term fluctuations.

Regarding the results of the inter-rater analysis, in overview, the ICCs values ranged from 0.967 to 0.994, in time-domain from 0.959 to 0.996, in frequency-domain from 0.796 to 0.987 and 0.885 to 0.987 in non-linear analysis (Fig. 2). The SEM values obtained through the analysis range from − 4.89 to 10.69% in overview variables, 0.72 to 8.83% in time domain, 2.47 to 38.05% in frequency domain and 1.55 to 10.35% in non-linear analysis. The MDC ranged from − 13.56 to 29.64% in overview, 1.91 to 24.47% in the time-domain, 6.84 to 105.47% in the frequency-domain and 4.30 to 28.69% in the nonlinear methods (Table 5). Regarding Bland–Altman plots of inter-examiner reliability, in the overview, the Stress Index exhibited a bias of 0.39 ms (Fig. 7). In the time domain, the largest variation was observed in Mean RR (− 0.22 ms) and TINN (− 7.67 ms) (Fig. 7). In the frequency domain, LF (7.64 ms^2^) and Total Power (12.14 ms^2^) showed substantial dispersion in the limits of agreement (Fig. 8). The nonlinear methods, SD1 (− 0.12 ms) and SD2 (− 0.42 ms), displayed minimal biases but greater variability (Fig. 8).

**Table 5:** Inter-rater reliability of HRV analysis of patients with TMD in the supine position.

HRV Index	SEM	SEM (%)	MDC	MDC (%)	CV (%)
Overview					
PNS Index	0.06	− 4.89	0.15	− 13.56	− 3.45
SNS Index	0.19	10.69	0.52	29.64	6.13
Stress Index	0.85	5.75	2.37	15.93	2.69
Time domain					
Mean RR (ms)	5.49	0.72	15.21	1.99	0.36
SDNN	1.53	4.80	4.25	13.30	2.47
Mean HR (bpm)	0.55	0.69	1.52	1.91	0.36
RMSSD (ms)	1.40	5.20	3.88	14.42	2.27
RR Tri	0.63	7.66	1.75	21.24	3.56
TINN	14.18	8.83	39.30	24.47	3.44
Frequency domain					
VLF (ms^2^)	31.56	38.05	87.49	105.47	14.88
LF (ms^2^)	100.32	15.85	278.07	43.93	8.54
HF (ms^2^)	73.69	18.91	204.26	52.41	6.43
VLF (log)	0.36	8.83	1.00	24.46	4.41
LF (log)	0.19	3.05	0.52	8.47	1.56
HF(log)	0.13	2.47	0.37	6.84	1.26
LF (n.u.)	3.76	5.72	10.42	15.87	3.18
HF (n.u.)	3.72	10.84	10.30	30.05	6.25
Total power	152.49	13.79	422.69	38.22	7.22
LF/HF	0.63	20.66	1.74	57.26	9.26
Nolinear methods					
SD1 (ms)	0.95	4.99	2.64	13.83	2.22
SD2 (ms)	2.00	4.93	5.55	13.67	2.64
SD1/SD2	0.08	3.53	0.23	9.79	1.97
Apen	0.02	1.55	0.05	4.30	0.81
SampEn	0.05	2.75	0.13	7.63	1.39
DFA α1	0.06	5.27	0.17	14.61	3.05
DFA α2	0.05	10.35	0.14	28.69	5.25

*HRV* heart rate variability, *SEM* standard error measurement, *MDC* minimal detectable change, *SD* standard desviation, *CV* coefficient of variation, *RR* interbeat intervals between all successive heartbeats, *PNS* parasympathetic nervous system, *SNS* sympathetic nervous system, *ms* milliseconds, *SDNN* standard deviation of the N–N interval, *bpm* beats per minute, *RMSSD* root mean square differences of successive RR intervals, *RR Tri* integral of the RR intervals histogram divided by the height of the histogram, *TINN* baseline width of the RR intervals histogram, *VLF* very-low frequency, *LF* low-frequency band, *HF* the high-frequency band, *ms*^2^: milliseconds squared, *n.u* normalized units, *log* logarithmic, *LF/HF* ratio of LF-to-HF, *SD1* Poincaré plot standard deviation perpendicular the line of identity, *SD2* Poincaré plot standard-deviation along the line of identity, *SD2/SD1* ratio of SD1-to-SD2, *ApEn* approximate entropy, *SampEn* sample entropy, *DFA α1* detrended fluctuations analysis. Which describes short-term fluctuations, *DFA α2* detrended fluctuation analysis. Which describes long-term fluctuations.

## Discussion

Our results demonstrate intra- and inter-examiner reliability of short-term resting HRV in individuals with TMD, whose ICC was ≥ 0.75 for all observed variables—independent of examiner and time of assessment. As such, this outcome confirms the hypothesis of the study and fills the gap presented in the introduction, as this is the first study on the reliability of HRV in patients with TMD.

Several studies have investigated musculoskeletal aspects in TMD patients, thus TMD is a musculoskeletal disorder categorized as intra-articular and/or extra-articular (involving the surrounding musculature)^30^. However, although TMD patients are known to suffer from autonomic dysfunction^5^, there are still no standardized autonomic characterizations to assess the nervous system of these patients (e.g., HRV parameters)^5^. We are aware that in order to evaluate HRV, it is first necessary to verify that the instrument and the evaluation method are reliable^14^, therefore, as a first in the area, our results support the possibility of lines of research on the autonomic profile of TMD patients since this measurement has adequate reliability.

A large retrospective study conducted by a single investigator over 25 years found that the most common signs and symptoms were facial pain, ear discomfort, headache, jaw discomfort/dysfunction, dizziness, neck pain, eye pain, arm pain, or back pain^31^; however, the authors did not discuss the neurological implications of TMD, highlighting the gap in this area of research. Namely, the study of nervous system function in TMD patients is still relatively new. Our study suggests an opportunity for research that brings together the fields of neuroscience and musculoskeletal dysfunction (e.g., HRV and chronic pain^32^). Thus, by understanding the autonomic and musculoskeletal characteristics of TMD patients, we will be able to propose better diagnoses and treatments for this population^30,33^.

Liu and Steinkeler^33^ described four goals of treatment for these patients: decrease joint pain; increase joint function and opening; prevent further joint damage; improve the overall quality of life and reduce disease-related morbidity. However, they did not recognize the importance of autonomic measurements in monitoring the prognosis. In contrast, Wieckiewicz et al.^34^ presented pharmacological and non-pharmacological treatment options, but none of the authors showed the importance of an acceptable evaluation of TMD patients. There are several treatment options in the literature (of course)^34^, but with limited evaluations, it is impossible to observe the results of the intervention. Therefore, we suggest that new studies expand the evaluation methods and verify the reliability of the tests and instruments used (as we did in this study). Because to know whether an intervention is effective, it is necessary to use a reliable assessment (i.e., ICC ≥ 0.75).

Although the exact mechanisms are not fully understood, it is known that chronic pain, frequently observed in individuals with TMD, is associated with alterations in the modulation of the autonomic nervous system^35,36^. These changes can be monitored through HRV. Chronic pain tends to favor an increase in sympathetic nervous system activity, which results in a reduction in HRV, indicating a lower capacity to adapt to physiological and emotional variations. HRV assessment offers a promising tool to investigate these phenomena and may contribute to the development of more effective therapeutic strategies for TMD treatment, as well as helping identify those at risk for unfavorable outcomes and cardiovascular complications^37^, regardless of the severity of TMD or the nature of the pain.

The strong intra- and inter-examiner reliability of HRV measurements supports its potential as a non-invasive biomarker for autonomic dysfunction in TMD patients. Clinically, HRV monitoring could help track disease progression, evaluate treatment outcomes, and guide personalized interventions. Furthermore, the use of wearable devices for continuous HRV monitoring opens the door for real-time, remote management, offering a futuristic approach to personalized healthcare in this patient population. Future studies may also explore HRV as a prognostic marker for TMD treatment outcomes, with reliable cut-off points helping to differentiate symptomatic from asymptomatic patients, aiding clinical decision-making and early interventions.

Regarding the limitations of this study, we performed the reliability analysis of the HRV analysis method recorded by a Polar cardio-frequency meter, model V800 (according to the previous studies^25,38–42^); however, this device is no longer available on the market. Therefore, our results may not be extrapolated to other samples and/or disorders due to biological individuality, as well as the specific characteristics of each disease. As such, we suggest that further studies investigate the same measurement properties in other diseases.

## Conclusion

The HRV analysis captured by a Polar cardio frequency meter presented adequate reliability when considering different times and examiners. The results indicate excellent intra- and inter-examiner reliability for HRV variables, with ICC values ranging from 0.842 to 0.999 for intra-examiner assessments and 0.796 to 0.994 for inter-examiner comparisons. The Bland–Altman plots confirmed good agreement, with minimal bias in most variables, although higher variability was observed, especially in the frequency domain. Moreover, the coefficients of variation were below 10% in all intra- and inter-examiner analyses, except for the VLF (ms^2^) variable. This exception may be attributed to the fact that VLF reflects more complex physiological mechanisms, including hormonal regulation and thermoregulatory processes, which are less directly controlled by autonomic modulation and more susceptible to external influences and methodological variations. Additionally, the VLF component is more susceptible to external factors and recording artifacts, making it more variable between measurements.

## Data Availability

The dataset produced and/or analyzed during the current study can be obtained from the corresponding author upon reasonable request.
